# Congenital lumbar and umbilical hernias in lumbo-costo-vertebral syndrome with ipsilateral renal agenesis: A rare case report with literature review

**DOI:** 10.1097/RC9.0000000000000244

**Published:** 2026-02-09

**Authors:** Layth J. M. Saada, Jamil Saada

**Affiliations:** a Faculty of Medicine and Health Sciences, Al-Quds University, Jerusalem, Palestine; b Pediatric Surgery Department, Al-Mezan speciality hospital, Hebron, West bank, Palestine; c Department of Surgery, Al-Quds University, Jerusalem, Palestine

**Keywords:** case report, congenital lumbar hernia, hernia mesh surgery, lumbo-costo-vertebral syndrome, meshplasty, pediatric surgery

## Abstract

**Introduction and importance:**

Congenital lumbar hernia (CLH) is a rare condition in infancy, often associated with lumbo-costo-vertebral syndrome (LCVS), which combines vertebral, rib, and abdominal wall anomalies. Genitourinary defects are uncommon and rarely documented.

**Case presentation:**

A 1-year-old girl presented with a left-sided CLH, small umbilical hernia, and prominent left anterior ribs protrusion manifesting as a chest wall bulge. Radiologic workup showed T10–T11 hemivertebrae, fused left laminae and ribs (10th–11th), scoliosis convex to the hernia side, and ipsilateral renal agenesis, confirming LCVS with a genitourinary anomaly. Elective open surgical repair included meshplasty of the left lumbar defect and excision of the protruding fused left anterior rib segment through the same incision, with concurrent umbilical hernia repair through a circumferential periumbilical incision. Recovery was uneventful.

**Clinical discussion:**

This case illustrates the syndromic nature of CLH within LCVS and underscores the need to screen for associated anomalies, particularly genitourinary defects such as renal agenesis. Imaging beyond ultrasound is often required to delineate skeletal and myofascial abnormalities. An open approach was favored to allow simultaneous excision of the protruding fused left rib segment and durable mesh reinforcement of a ~5 cm defect; this strategy achieved excellent outcomes. The coexistence of umbilical hernia with LCVS and CLH may be incidental but could broaden the recognized phenotypic spectrum and, to our knowledge, has not been previously reported.

**Conclusion:**

Awareness of LCVS in infants with CLH facilitates evaluation for associated anomalies and guides timely surgical management. Early recognition and repair can yield favorable results.

## Introduction

Congenital lumbar hernia (CLH) is a rare congenital condition that results from a defect in the abdominal wall, with only around 85 cases reported since 2000^[^1^]^. It may present as an isolated anomaly or in association with other congenital abnormalities, most commonly as a part of lumbo-costo-vertebral syndrome (LCVS)^[^2^]^. As the name indicates, LCVS is characterized by combined malformations of the abdominal wall musculature, ribs (including absence, hypoplasia, fusion, or gaps), and the vertebral column (such as hemivertebrae, scoliosis, or vertebral defects). CLH typically presents as a painless, soft, and reducible mass in the lumbar region that becomes more pronounced with crying or straining, and most of them occurs through Grynfeltt’s superior lumbar triangle, while the inferior triangle of Petit is less commonly involved. While many cases of CLH are asymptomatic and manifest only as visible swelling, larger defects or associated anomalies may cause symptoms such as lower back pain or discomfort, which is the most frequently reported complaint in affected patients ^[^3^]^. LCVS is usually present during infancy or early childhood^[^4^]^ and is often associated with other congenital anomalies. Due to its rarity and varied presentation, lumbar hernias in the context of LCVS have occasionally been misdiagnosed as lipomas^[^5,6^]^. Diagnosis can be challenging and often requires a high index of suspicion along with comprehensive imaging. Here, we report a unique case of LCVS in a 1-year-old Palestinian girl presenting with a left lumbar hernia, small umbilical hernia, prominent disfiguring protrusion of the left lower ribs, and ipsilateral renal agenesis – underscoring the importance of early recognition and multidisciplinary management of this rare condition.

This case report has been reported in line with the SCARE 2025 criteria^[^7^]^.

HIGHLIGHTS

- Congenital lumbar hernia (CLH) is a rare entity in infancy, frequently associated with lumbo-costo-vertebral syndrome (LCVS).
- This case demonstrated CLH with multiple anomalies, including hemivertebrae, rib fusion, scoliosis, and ipsilateral renal agenesis.
- Elective repair with meshplasty, excision of the prominent segment of the fused left lower ribs, and concurrent umbilical hernia repair achieved excellent recovery.
- The coexistence of umbilical hernia with LCVS and CLH may be incidental but could broaden the recognized phenotypic spectrum and, to our knowledge, has not been previously reported.

## Case presentation

**Figure 1.**
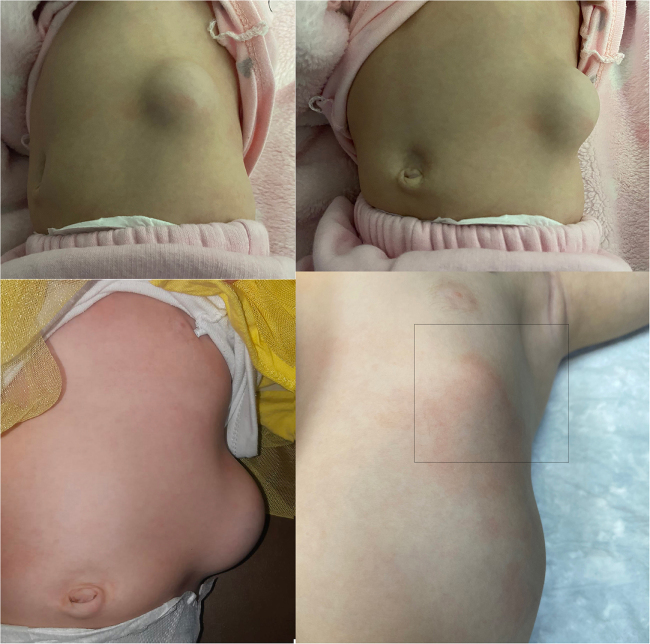
Clinical photographs of a 1-year-old girl showing a left lumbar hernia and a subtle external chest wall bulge corresponding to the prominent edge of the left lower ribs (rectangle). A small umbilical hernia was noted on examination, although it is not clearly visible in these images.

A 1-year-old girl presented to the pediatric surgery department with a left lumbar hernia, a small umbilical hernia, and a prominent edge of the left lower ribs, located just below the nipple, laterally along the anterior axillary line (Fig. 1). These findings have been apparent since birth. Her birth history was unremarkable; she was delivered via cesarean section at term with a birth weight of approximately 3.2 kg. At admission, her weight was around 11 kg, and her growth parameters were within normal limits for age.

There was no history of respiratory distress, recurrent chest infections, or feeding difficulties during infancy. The patient appeared well and in no distress. Clinical examination revealed a small, reducible umbilical hernia with a positive cough impulse. In the left lumbar region, a soft, fluctuant, and reducible globular swelling measuring approximately 6 × 7 cm was palpated, also with a positive cough impulse. Bowel sounds were audible over the mass. There were no signs of tenderness, incarceration, or overlying skin changes. Neurological reflexes, cardiac examination, and anorectal assessment were all unremarkable. Routine laboratory investigations were within normal limits.

**Figure 2.**
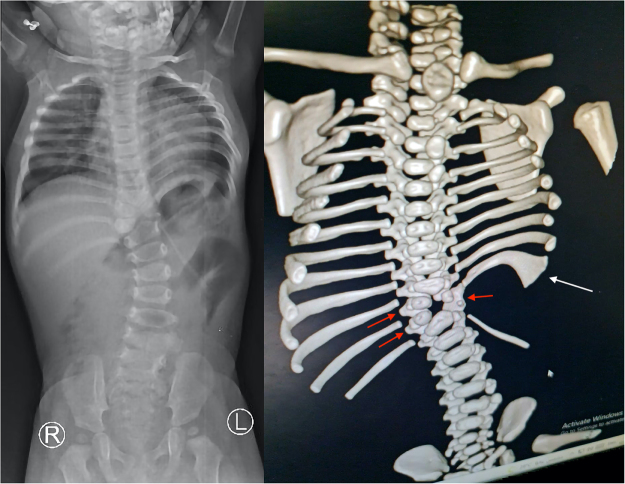
3D CT mainly showing skeletal anomalies, including hemivertebrae at T10 and T11 (red arrows), fused laminae at these levels, congenital fusion of the left 10th and 11th ribs (white arrow), and scoliosis convex toward the side of the lumbar hernia.

Abdominal ultrasound suggested a lumbar hernial defect measuring roughly 5 × 6 cm containing bowel loops, along with left renal agenesis. CT scan with 3D reconstruction confirmed the lumbar hernial defect, clearly demonstrated the absence of the left kidney, and additionally showed vertebral anomalies, including hemivertebrae at T10 and T11, fusion of the left laminae at these levels, congenital fusion of the left 10th and 11th ribs, and scoliosis with a convex curve on the side of the lumbar hernia (Fig. 2). These findings were accompanied by significant narrowing of the left intercostal spaces and a prominent anterior protrusion of the left lower ribs, resulting in a noticeable asymmetry and discrepancy of the shape of the anterior chest wall. Based on this constellation of findings, a diagnosis of LCVS was established. Echocardiography demonstrated no structural cardiac abnormalities.

**Figure 3.**
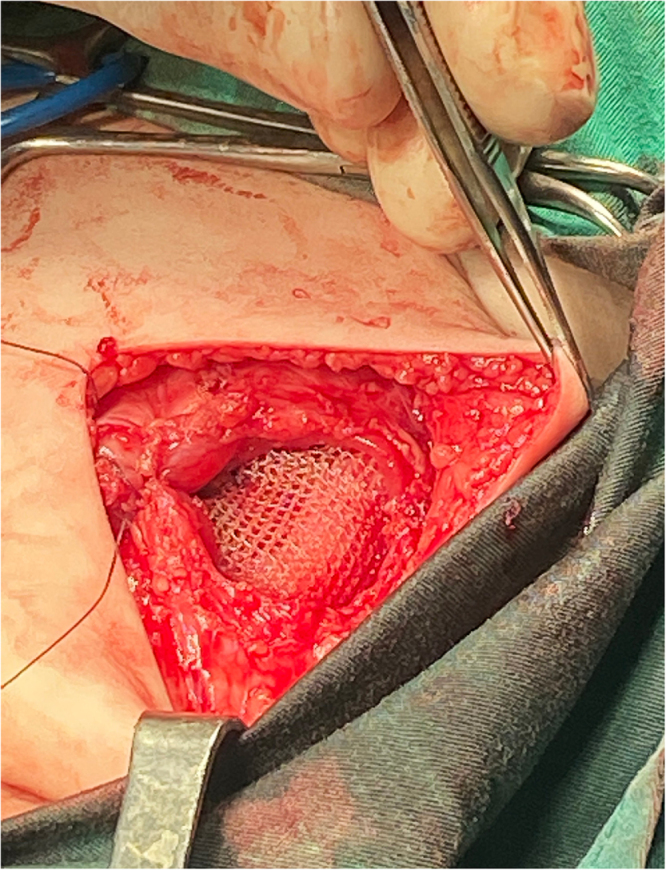
Intraoperative view; mesh in place covering the defect.

Elective open surgical repair was performed under general anesthesia. The procedure involved excision of the anterior protruding segment of the fused left 10th–11th ribs, meshplasty of the left subcostal muscular wall defect measuring approximately 5 cm in diameter through the same incision (Fig. 3), and concurrent repair of the umbilical hernia performed through a circumferential periumbilical incision.

The postoperative course was uneventful, with stable vital signs and appropriate recovery parameters throughout the day. The procedure was performed in the morning, and the patient was discharged approximately 15 hours later at the parents’ request and under their responsibility. Long-term orthopedic follow-up was advised to monitor and manage any potential functional impairments associated with LCVS. Early postoperative follow-up within the first few days was unremarkable. Subsequent evaluations at intervals of several months, for a period of up to three years, demonstrated a satisfactory recovery without complications or recurrence.

The clinical course is summarized as follows:

Birth: Lumbar swelling, subtle left lower ribs prominence, and umbilical bulge noted since birth.

Age 1 year: presentation to the pediatric surgery clinic with a left lumbar hernia, a small umbilical hernia, and a rib/vertebral deformity confirmed clinically and radiologically.

Preoperative period: CT with 3D reconstruction revealed vertebral, rib, and abdominal wall anomalies consistent with LCVS, along with left renal agenesis.

Surgery: elective open repair was performed with meshplasty of the lumbar defect, excision of the anterior protruding segment of the fused left 10th–11th ribs through the same incision, and concurrent repair of the umbilical hernia.

Early postoperative period (within the first week): stable recovery with no complications.

Subsequent follow-up: evaluated periodically at several-month intervals for up to 3 years, with no recurrence or functional concerns.

## Discussion

Lumbar hernia (LH) is an extremely rare entity, representing less than 1.5% of all abdominal wall hernias^[^8^]^. It typically arises in the posterolateral region of the abdominal wall, where various structures – such as bowel loops, omentum, ovary, spleen, or kidney – can protrude through one of the lumbar triangles. Thorek classified LHs based on their contents into extraperitoneal (lacking a peritoneal hernial sac), paraperitoneal, or intraperitoneal types^[^9^]^. Lumbar masses in infants may resemble a wide range of benign lesions; therefore, establishing an accurate differential diagnosis is essential before confirming a CLH. Its rarity often leads to misdiagnosis as benign soft-tissue lesions such as lipoma, cold abscess, fibromas, or benign and malignant soft-tissue tumors, as well as hematomas, seromas, and renal tumors, potentially resulting in delayed recognition and management^[^5,6^]^. These hernias have been categorized based on etiology as either congenital (about 20%) or acquired (80%), with most acquired cases (55%) being primary, while 25% are secondary, often resulting from trauma, surgery, or inflammation^[^10^]^. This classification highlights CLH as the rarest variant. The existence of LH was first proposed by Barbette in 1672, and the first case was reported by De Garengeot in 1731^[^11,12^]^. Further anatomical insights were later contributed by Petit in 1783 – who described the inferior lumbar triangle – and by Grynfeltt in 1866, who described the superior lumbar triangle, both recognized as the primary anatomical sites for LH development.

LHs can be anatomically classified into three types; superior, inferior, and diffuse lumbar hernias. The superior lumbar triangle, also known as Grynfeltt-Lesshaft’s triangle, is bordered superiorly by the 12th rib, medially by the erector spinae muscles, and laterally by the posterior border of internal abdominal oblique muscle. Its roof is formed by the latissimus dorsi muscle, while the floor consists of the transversus abdominis fascia. The inferior lumbar triangle, or Petit’s triangle, is bounded medially by the lateral border of the latissimus dorsi muscle, laterally by the posterior border of the external oblique muscle, and inferiorly by the iliac crest, which forms its base. The floor of the triangle is formed by the relatively thin internal oblique muscle, while the roof consists of the superficial lumbodorsal fascia, skin, and subcutaneous tissue. In some cases, lumbar hernias may occur diffusely, developing outside these classical lumbar triangles anywhere within the lumbar region, as has been documented in the literature^[^10,13^]^.

CLH often occurs with vertebral and rib anomalies, and when present together, this defines LCVS – a rare congenital disorder with diverse anatomical abnormalities of the abdominal wall, ribs, and vertebral column^[^2^]^. It is often associated with additional congenital abnormalities, including renal, spinal, and musculoskeletal defects. Previously reported associations include vertebral segmentation defects (such as hemivertebrae and scoliosis), rib abnormalities (including absence, fusion, or hypoplasia), abdominal wall hypoplasia, and genitourinary anomalies, including renal agenesis. Although very rare, LCVS may also be found in conjunction with VACTERL association, including spinal, cardiac, renal, gastrointestinal, and limb anomalies^[^14^]^. The association between CLH and LCVS is sparsely reported in the literature^[^15^]^. Although the exact pathogenesis of LCVS remains uncertain, it was first described by Touloukian in 1972, who proposed the embryologic origin hypothesis – suggesting that a single somatic defect, possibly resulting from transient anoxia during the third to fifth weeks of gestation, may impair mesodermal development, leading to simultaneous malformations of the vertebrae, ribs, abdominal wall musculature, and occasionally the genitourinary system^[^16^]^.

In our case, the presence of T10–T11 hemivertebrae, fusion of the left laminae at these levels, congenital fusion of the left 10th and 11th ribs, scoliosis with the convex curve on the site of hernia, abdominal wall hypoplasia, and left renal agenesis is consistent with the proposed embryologic explanation. Notably, the renal agenesis contributes to the distinctiveness of this presentation.

To date, reports describing the coexistence of CLH, LCVS, and ipsilateral renal agenesis remain extremely limited. Furthermore, the patient presented with a small umbilical hernia, a relatively common and often self-resolving condition in the pediatric population. However, its occurrence alongside more complex anomalies – such as CLH and vertebral/rib malformations seen in this case – may possibly indicate an underlying syndromic or developmental association, though it could also be an incidental finding. To the best of our knowledge, the coexistence of an umbilical hernia with LCVS and CLH has not been previously reported in the literature. This rare combination of findings highlights the uniqueness of the case and may expand the phenotypic spectrum associated with LCVS. By detailing this association and the comprehensive musculoskeletal and genitourinary anomalies, this report contributes new insight into the spectrum and embryologic implications of LCVS-related defects.

The anatomical location of the hernia can also be interpreted in light of existing hypotheses. Hernias protruding through Grynfeltt’s triangle – the superior lumbar triangle – are the most common overall, especially in spontaneous and congenital cases, and tend to be larger and deeper^[^8,13^]^. Our case aligns with this pattern, as the hernia was located in the superior lumbar triangle (Grynfeltt’s). This area is more susceptible to hernia formation due to a natural defect in the transversalis fascia, where the 12th dorsal neurovascular bundle passes. Such inherent anatomical vulnerability creates a weak point that predisposes to abdominal content protrusion^[^13^]^. Along with the embryologic origin hypothesis, these factors provide a plausible framework for understanding the predominance of congenital and primary lumbar hernias at this site. Conversely, hernias occurring through Petit’s triangle – the inferior lumbar triangle – are usually traumatic in origin, due to the thinner muscular floor, superficial position, and greater exposure to blunt abdominal trauma. This mechanism accounts for the predominance of traumatic hernias at this site, as reported by Malik et al. in a small case series, where approximately 70% of traumatic lumbar hernias occurred through the inferior triangle^[^10^]^.

CLH has variable clinical features. Characterizing its typical age at presentation, gender distribution, and laterality is crucial for improving diagnosis and management. Rattan *et al*, in a case series of 14 patients, reported that all children with CLH presented within the first two years of life. Similarly, Sharma *et al* observed a median presentation age of 3 months. Tasis *et al* also reported a mean age of 9.7 ± 20.7 months, suggesting a skewed distribution toward infancy with few outliers at older ages. These findings collectively indicate that most CLH cases tend to be present within the first 2 years of life, typically during infancy. Regarding gender, Tasis *et al* found a slight male predominance (55.7%), while Rattan *et al* reported a stronger male predominance (86%), and Sharma *et al* found no significant gender variation^[^1,2,17^]^. Regarding laterality, Rattan *et al* observed a predominance of right-sided hernias (11 right: 2 left: 1 bilateral), whereas Tasis *et al*, in a systematic review of 85 cases, reported a slight left-sided predominance, with a distribution ratio of approximately 5:4:1 for left/right/bilateral presentations, respectively^[^1,2^]^. Bilateral hernias were the rarest in these three studies. Further inconsistencies exist in the literature regarding the most affected lumbar triangle. These variations underscore the value of documenting each case to enhance clinical awareness and broaden the available data.

Clinical suspicion is key, as herniated fat can mimic a lipoma. While ultrasound is often used as an initial imaging tool, CT or MRI is typically required to confirm the diagnosis and assess defect size, contents, and the anatomy of disrupted muscle layers^[^18^]^. In our case, CT scan with post-processing 3D reconstruction provided a comprehensive view of the hernia and associated musculoskeletal anomalies. CLH requires timely surgical intervention to avoid complications like obstruction or strangulation. Small defects under 5 cm can often be closed primarily with tissue approximation, whereas larger defects usually necessitate mesh reinforcement to restore a durable and flexible abdominal wall. Both open and laparoscopic approaches are viable^[^2,19^]^, with the choice depending on specific indications and surgical challenges. Due to the hernia size and associated rib deformity in this case, an open surgical approach is preferred. It allows better access for effective hernia repair and simultaneous excision of the prominent parts of the disfiguring ribs, which is difficult to address laparoscopically, ensuring better outcomes.

## Conclusion

Although LCVS is a rare anomaly, awareness is essential to prevent misdiagnosis. This case highlights the unique association of CLH and ipsilateral renal agenesis, along with an umbilical hernia, which may represent an incidental finding or a possible extension of the LCVS spectrum, potentially expanding the known phenotypic spectrum of the condition. Early recognition, comprehensive imaging using ultrasound and CT, and timely surgical intervention are crucial for achieving optimal outcomes. Reporting such rare cases contributes valuable knowledge for improved diagnosis and management.

## Data Availability

All data supporting this case report are included within the article, and additional information can be provided upon reasonable request.

## Research registration unique identifying number (UIN)

Research registration was not applicable for this case report.

## References

[1] Tasis N Tsouknidas I Antonopoulou MI. Congenital lumbar herniae: a systematic review. Hernia 2022;26:1419–25. 34347187 10.1007/s10029-021-02473-x

[2] Rattan KN Agarwal A Dhiman A. Congenital lumbar hernia: a 15-year experience at a single tertiary centre. Int J Pediatr 2016;2016:7162475. 27994626 10.1155/2016/7162475 PMC5138478

[3] Eshetu B Mekonnen T Berhane M. Lumbo-Costo-Vertebral syndrome with congenital lumbar hernia: case report. Ethiop J Health Sci 2019;29:413–16. 31447511 10.4314/ejhs.v29i3.15 PMC6689718

[4] Geis WP Hodakowski GT. Lumbar hernia. In: Nyhus LM Cohen RE, eds. Nyhus and Condon’s The Hernia. Philadelphia: JB Lippincott; 1995:416–18.

[5] Ahmed ST Ranjan R Saha SB. Lumbar hernia: a diagnostic dilemma. Case Rep 2014;2014:bcr2013202085. 10.1136/bcr-2013-202085 PMC3992605 24810439

[6] Tayeb M Abbassi A El Hassani AE. Lumbar hernia: a commonly misevaluated condition of the bilateral costoiliac spaces. Trauma Case Rep 2017;12:22–26.

[7] Kerwan A Al-Jabir A Mathew G. Revised Surgical CAse REport (SCARE) guideline: An update for the age of Artificial Intelligence. Prem J Sci 2025;10:100079.

[8] Sharma Lt Col P. Lumbar hernia. Int J Surg Case Rep 2009;1:30–32.

[9] Thorek M. Modern surgical technique. Philadelphia. JB Lippincott; 1950:23–32.

[10] Malik DS Dhakad BS Singh M. Traumatic lumbar hernia: a series of three cases. Peer Educ J 2022;2:45–49.

[11] Barbette P. Opera chirurgico-anatomica. Lugduni: J a Gelder. 1972;26:1672.

[12] De Garangeot RJC. Traite des operations de chirurgie. Teratology 1731;1:369.

[13] Moreno-Egea A Baena EG Calle MC. Controversies in the current management of lumbar hernias. Arch Surg 2007;142:82–88. 17224505 10.1001/archsurg.142.1.82

[14] Harris K Dorn C Bloom B. Lumbocostovertebral syndrome with associated VACTERL anomalad: a neonatal case report. J Perinatol 2009;29: 826–27. 19935732 10.1038/jp.2009.82

[15] Kumar GS Kulkarni V Haran RP. Lumbo-costo-vertebral syndrome with posterior spinal dysraphism. Neurol India 2005;53: 351–53. 16230812 10.4103/0028-3886.16943

[16] Touloukian RJ. The lumbocostovertebral syndrome: a single somatic defect. Surgery 1972;71:174–81. 5057828

[17] Sharma A Pandey A Rawat J. Congenital lumbar hernia: 20 years’ single centre experience. J Paediatr Child Health 2012;48: 1001–03. 23039934 10.1111/j.1440-1754.2012.02581.x

[18] Killeen KL Girard S DeMeo JH. Using CT to diagnose traumatic lumbar hernia. AJR Am J Roentgenol 2000;174: 1413–15. 10789805 10.2214/ajr.174.5.1741413

[19] Gupta L Mala TA Gupta R. Lumbo-costo-vertebral syndrome with congenital lumbar hernia. APSP J Case Rep 2014;5:5. 24834386 PMC4005092

